# Innovative Production of Diclofenac Sodium-Loaded Solid Lipid Nanoparticles by Membrane Nanoprecipitation: Evaluation of Encapsulation Efficiency and Photoprotection Ability

**DOI:** 10.1007/s11095-026-04139-8

**Published:** 2026-06-19

**Authors:** Fabio Bazzarelli, Giuseppina Ioele, Martina Chieffallo, Lidietta Giorno, Antonio Garofalo, Alessio Fuoco, Emma Piacentini

**Affiliations:** 1 National Research Council of Italy, Institute On Membrane Technology “Enrico Drioli” (CNR-ITM), Via P. Bucci, 17/C, 87036 Rende, Italy; 2 Department of Pharmacy, Health and Nutritional Sciences, University of Calabria, 87036 Rende, Italy

**Keywords:** Diclofenac sodium, Membrane nanoprecipitation, Photodegradation, Solid lipid nanoparticles

## Abstract

**Purpose:**

The stability of diclofenac sodium (DS) in liquid formulation is influenced by environmental factors such as temperature, humidity and light. The encapsulation of this drug in solid lipid nanoparticles (SLNs) can offer a useful strategy to protect it from degradation. The present study shows the potential of membrane nanoprecipitation (MN) as a productive and advantageous method for the formulation of cocoa butter-based SLNs to improve the photostability of DS.

**Methods:**

Conventional batch nanoprecipitation and MN were explored by varying the formulation parameters and process conditions to obtain SLNs.

**Results:**

The MN produced SLNs with an average size of 421 ± 14 nm, a high encapsulation efficiency of 95 ± 3.2%, and a drug loading of 8.8 ± 0.2%, when an acetate buffer at pH 4.1 was used as the non-solvent phase. Chemical characterization of the proposed formulation was performed by DSC and FTIR analyses. The efficacy of SLNs in preserving DS stability was investigated under simulated sunlight exposure, demonstrating a residual drug concentration of 90% after 3.34 min with respect to the aqueous solution that showed the same concentration value after 0.072 min.

**Conclusions:**

Overall, MN represents a suitable and sustainable process for the continuous production of light-stable drug-loaded SLNs.

## Introduction

Solid lipid nanoparticles (SLNs) are increasingly being investigated as carriers for protecting sensitive active ingredients from environmental factors and for designing drug delivery systems. SLNs, which range in size from 50 to 1000 nm, are used in various fields (e.g., medical, pharmaceutical, cosmetic, and nutraceutical) as alternative drug carriers to liposomes, emulsions and polymeric particles. The use of lipid carriers offers many advantages, such as lower risk of toxicity, better cellular absorption, and the ability to encapsulate both hydrophilic and hydrophobic molecules [1, 2]. Several types of solid lipids at room temperature, including triglycerides, fatty acids, fatty alcohols and waxes, are used to produce SLNs. Natural lipids such as cocoa butter (CB) offer a sustainable alternative to synthetic lipids for the formulation of SLNs. CB is a vegetable fat extracted from *Theobroma cacao* beans, which is composed mainly of triglycerides with stearic and palmitic acids as the principal saturated fatty acids and oleic acid as the major unsaturated fatty acid [3].

CB is conventionally used in the chocolate and confectionery industries, as well as in the production of ointments for its moisturizing and emollient properties. It is also used in the development of drug delivery systems [4] and several molecules have been encapsulated in CB-based carriers [4–11]. Its use as a pharmaceutical excipient is supported by approval from the U.S. Food and Drug Administration (FDA) and the European Medicines Agency (EMA), owing to its good biocompatibility and lower in vivo toxicity relative to several semi-synthetic lipid alternatives [12].

SLNs are commonly produced by high-pressure homogenization, solvent emulsification (evaporation or diffusion), ultrasonication, microemulsion and nanoprecipitation (also referred to as solvent displacement or solvent injection) [13, 14].

Nanoprecipitation is a simple method involving the mixing of two miscible liquids, one in which the solute is dissolved (solvent phase) and one in which it is insoluble (non-solvent phase). The addition of the solvent containing the solute to the non-solvent induces supersaturation of the solute, which leads to nucleation and growth of particles.

In recent decades, nanoprecipitation has been performed in combination with membrane technology to potentially scale up nanoparticles (NPs) production from the laboratory to the industrial scale [15–17]. In the membrane nanoprecipitation (MN) process, a porous membrane is used to compartmentalize the solvent and non-solvent phases and serves as an efficient and precise liquid-dispersion system to control their mixing.

Compared to conventional methods, MN is a low-energy process that does not require high temperatures or elevated operating pressures. It is widely used due to its simplicity, scalability, and ability to produce particles with controlled sizes from a wide variety of materials (e.g., polymers, drugs, and lipids) [16].

Although the use of MN to produce liposomes has been reported in several studies [18, 19], to the best of our knowledge, its application in the production of SLNs has not yet been systematically investigated.

The pharmaceutical industry has been gaining attention for its environmental footprint due to intensive solvent consumption and the waste generation per unit of product. Processing and disposing of such large amounts of waste have a substantial financial impact, as well as the energy required to maintain plant productive operations [20]. Combining the advantages of the MN method with the use of natural lipids represents a possible green solution for the development of drug delivery systems based on lipid carriers. Following the pioneering study on the use of the solvent injection method for manufacturing SLNs with a variety of lipid matrices (from low to high melting points) [13], the production of CB-based SLNs by MN was attempted for the first time with the aim of designing SLNs for drug encapsulation and photoprotection in topical formulations.

The drug stability within SLNs is well documented and solid lipid matrices can effectively trap and hold drug molecules, creating a physical barrier that shields them from external factors such as light and oxygen. This shielding effect prevents the drug from being exposed to photodegradation processes. Among lipid components, CB inherently possesses properties that absorb or reflect UV radiation and when formulated into NPs, it can act as an integral part of the protective layer [8]. It is notable for its rich composition of fatty acids and cholesterol but also tocopherol that is essential for its antioxidant properties, which help in maintaining matrix quality and health benefits [4, 21], while mitigating UV-induced degradation of both the lipid matrix and photosensitive drugs.

Moreover, CB offers moisturizing and skin-conditioning benefits that can enhance transdermal drug absorption. The prepared CB-based SLNs have the potential for topical skin application. The melting point of CB is close to body temperature, which may facilitate drug absorption whilst providing a moisturizing and skin-conditioning effect. DS was selected as a model drug to define its complete photodegradation profile when loaded into CB-based SLNs.

In our previous studies, photostability of DS, a non-steroidal anti-inflammatory drug commonly used for the treatment of arthritis [22], has been tested in different conditions. The high sensitivity to light of DS has been demonstrated in methanol solution, standard gel and commercial gel specialty. On the contrary, very high stability has been measured when the antioxidant molecules or UV-absorber agents have been incorporated in DS gel or DS has been entrapped in cyclodextrins or niosomes [23, 24]. Lipid-based carriers (e.g., SLNs, liposomes, transferosomes, and ethosomes) have also been studied in the literature for the encapsulation of DS to enhance its transdermal and corneal permeation [25–28].

The nanoprecipitation conditions that permit the production of CB-based SLNs have not been previously studied, and a preliminary study was carried out using conventional batch nanoprecipitation to determine the effects of lipid concentration and solvent/non-solvent volumetric ratio on CB nanoprecipitation.

To achieve a continuous production of CB-based SLNs, the MN process was studied by compartmentalizing the two phases in the membrane module (with the non-solvent in the shell and the solvent in the lumen, and vice versa). The effect of some chemical parameters (lipid concentration, solvent/non-solvent volumetric ratio) and operative conditions (flux through the membrane, shear stress) on particle size and particle size distribution was investigated.

The main challenge in the production of drug-loaded particles is to achieve high drug encapsulation. This reduces waste generation due to free drug, lowers formulation costs, and minimizes the amount of drug-loaded carrier needed for administration.

NPs produced by nanoprecipitation often have a low drug loading (less than 10 wt%) due to the significant difference in the precipitation time between the drug and lipid materials [29, 30]. Herein, a strategy was used to improve the EE of DS in SLNs through nanoprecipitation by varying the pH of the non-solvent phase, which enabled the precipitation of the drug first, followed by the lipid.

Moreover, the suitability of the developed formulation to enhance the photostability of the drug was evaluated and compared with that of the DS solution.

Photodegradation experiments were made according to the International Conference on Harmonization (ICH) rules [31], while photodegradation kinetics was calculated by using chemometric methods. At this aim, the algorithm Multivariate Curve Resolution-Alternating Least Squares (MCR-ALS) was selected for its ability in estimating the pure spectra and the concentration (time) profiles of the drug and relative photoproducts [32–34].

## Materials and Methods

### Materials

Pure CB was purchased from a local market (Rende, Italy). Absolute ethanol, methanol and DS were supplied by Sigma-Aldrich (Milan, Italy). Acetic acid, sodium acetate, sodium dihydrogen phosphate, and disodium hydrogen phosphate were obtained from VWR International (Milan, Italy). Deionized water was produced using the Elix system (Millipore). All the materials were used as received, without further purification.

### Preparation of SLNs by Conventional Batch Nanoprecipitation

CB was dissolved in ethanol in the concentration range from 0.1 to 1% (w/v) at 40 ± 2 °C. The CB solution in ethanol served as the solvent phase, while water was employed as the non-solvent phase in the preparation of SLNs at room temperature (25 ± 2 °C). The CB solution was added dropwise to 10 mL of deionised water under magnetic stirring (250 rpm) to achieve a solvent/non-solvent volumetric ratio in the range from 0.1 to 0.8 (v/v). The same procedure was followed to prepare DS-loaded SLNs, using a solution of 0.1% w/v DS and 1% w/v CB in ethanol as the solvent phase and pure water or acetate buffer (pH 4.1) as the non-solvent phase. The lipid formulations were prepared with a solvent/non-solvent volumetric ratio of 0.2.

### Production of SLNs by Membrane Nanoprecipitation

**Fig. 1:**
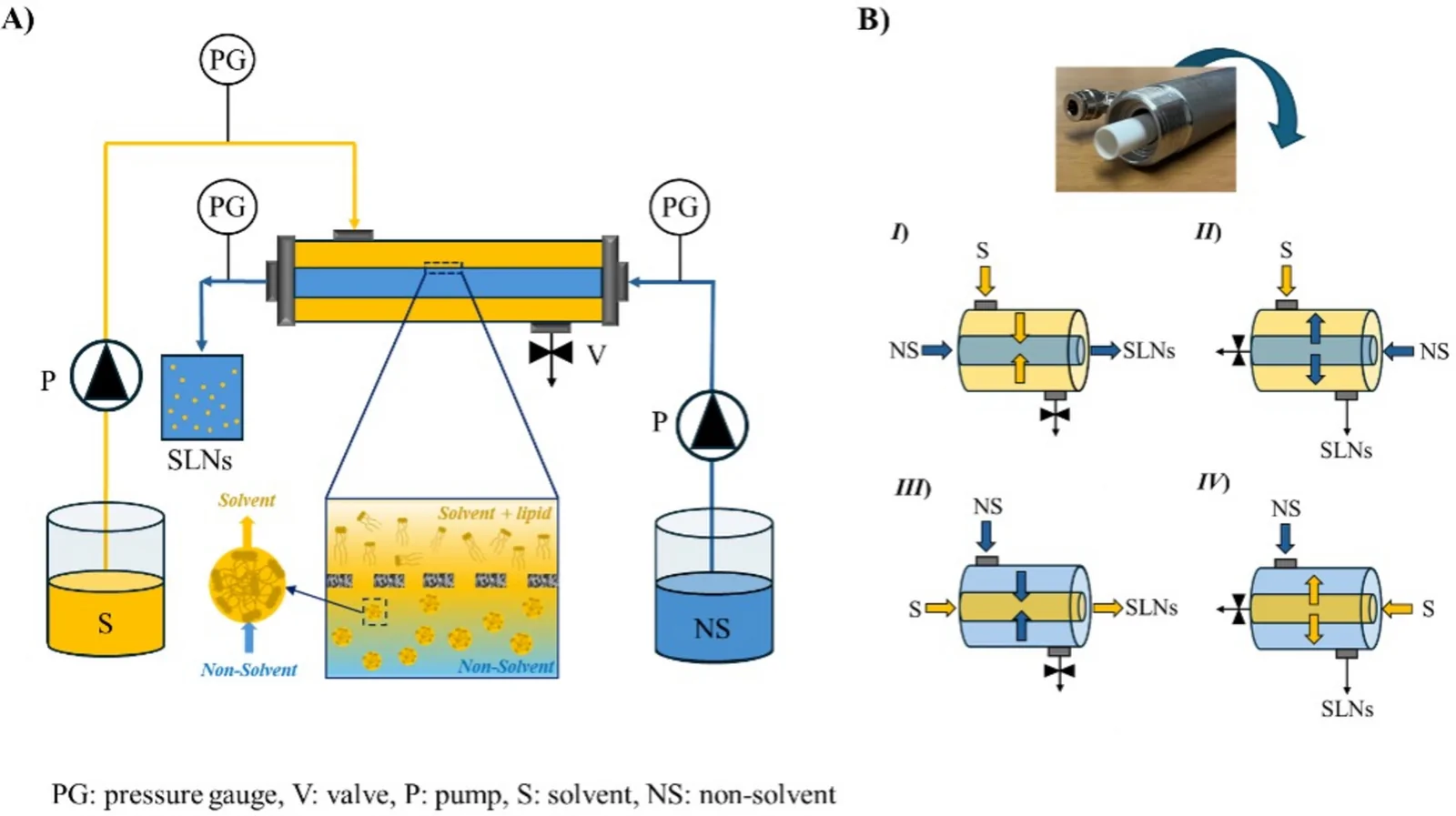
Schematic representation of the MN setup **A**; MN configurations **B**

The CB solution, prepared as described above, was employed for the continuous production of SLNs by the MN process, which was carried out using a laboratory-scale setup (Fig. 1a). The system consisted of a stainless-steel membrane module to accommodate a tubular hydrophilic Shirasu Porous Glass (SPG) membrane (SPG Technology, Miyazaki, Japan), with a pore size of 1 μm and an active surface area of 31.3 cm^2^. The experimental setup also included two peristaltic pumps (Masterflex® L/S, Cole-Parmer, Instrument Company, Quebec, Canada), one for feeding the CB solution and the other for feeding the aqueous phase into the membrane module. The two solutions flowed simultaneously, one along the lumen side of the membrane and the other along the shell side in a single-pass cross-flow mode. SLNs were collected at the outlet of the membrane module. The CB solution permeated under a pressure gradient through the membrane pores into the non-solvent (aqueous phase) or vice versa, according to the following MN configurations (Fig. 1b):

I. The solvent was fed on the shell side and permeated into the non-solvent flowing along the lumen side.
II. The non-solvent was fed on the lumen side and permeated into the solvent flowing along the shell side.
III. The non-solvent was fed on the shell side and permeated into the solvent flowing along the lumen side.
IV. The solvent was fed on the lumen side and permeated into the non-solvent flowing along the shell side.

The flow rates of the pumps were adjusted to achieve a solvent/non-solvent volumetric ratio between 0.2 and 1.25 (v/v), using a CB concentration of 0.1% (w/v).

The effect of the flow rate on particle size and particle size distribution was studied at a solvent/non-solvent volumetric ratio of 0.8, varying the organic phase flow rate between 15.2 and 104 mL min⁻^1^, corresponding to organic flux from 288 to 1994 L h⁻^1^ m⁻^2^. To maintain the solvent/non-solvent volumetric ratio of 0.8, the flow rate of the aqueous phase flowing tangentially to the membrane surface was varied from 19 to 130 mL min⁻^1^, resulting in a change in the maximum shear stress from 0.005 Pa to 0.032 Pa. The maximum shear stress was calculated using the equation provided by Koris et al. [35].

The DS-loaded SLNs were produced using a solvent phase consisting of DS and CB dissolved in ethanol at concentrations of 0.1% w/v and 1% w/v, respectively. Pure water or acetate buffer (50 mM, pH 4.1) served as a non-solvent phase. The SLNs were prepared with organic and aqueous phase flow rates of 31 and 155 mL min⁻^1^, respectively (solvent/non-solvent volumetric ratio: 0.2; shear stress of 0.040 Pa). After each experiment, the SPG membrane was cleaned in water at 40 °C using an ultrasonic bath to restore its initial performance.

The greenness of the MN process was assessed by evaluating the simple E-factor (sEF), complete E-factor (cEF), %cEF, %solvent + water, and energy consumption, according to the equations reported in our previous work [36].

To recover and purify the SLNs, the formulation was subjected to ultrafiltration (UF) using a centrifugal filtration device (Vivaspin®) equipped with a regenerated cellulose membrane having a molecular weight cut-off (MWCO) of 30 kDa. Centrifugation was performed at 5,000 rpm for 5 min. The procedure was repeated until the solvent mixture was completely recovered on the permeate side, while the wetted SLNs were collected on the retentate side.

### Determination of Encapsulation Efficiency and Drug Loading

The encapsulation efficiency of DS was evaluated by an indirect method using UV–VIS spectrophotometry. The encapsulation efficiency (EE) and drug loading (DL) were then calculated according to the following equations:

$$\mathrm{E}\mathrm{E} \left(\mathrm{\%}\right)=\left(\frac{{\mathrm{M}}_{\mathrm{i}}{-\mathrm{M}}_{\mathrm{f}}}{{\mathrm{M}}_{\mathrm{i}}}\right) \mathrm{x} 100$$

$$\mathrm{D}\mathrm{L} \left(\mathrm{\%}\right)=\left(\frac{{\mathrm{M}}_{\mathrm{D}\mathrm{S}}}{{\mathrm{M}}_{\mathrm{D}\mathrm{S}-\mathrm{S}\mathrm{L}\mathrm{N}\mathrm{s}}}\right) \mathrm{x} 100$$

where M_i_ and M_f_ represent the initial mass of DS and the mass of free (unencapsulated) DS, respectively. M_DS_ is the mass of encapsulated DS and M_DS-SLNs_ is the total mass of DS-loaded SLNs.

The M_f_ was determined into the permeate obtained by centrifuging the lipid formulation according to the procedure previously described. The permeate solution was analyzed using a spectrophotometer (Perkin-Elmer Lambda EZ, Waltham, MA, USA) at 272 nm and considering a calibration curve prepared from standard DS solutions with concentrations ranging from 2 to 30 mg/L.

### Characterization of SLNs

The mean particle size (Z-Average), zeta potential, and polydispersity index (PDI) of SLNs were determined using dynamic light scattering (Zetasizer Nano ZS, Malvern Instruments, Grovewood Road, United Kingdom). Measurements were performed in triplicate on lipid nanoparticle formulations diluted in deionized water, which was selected as the dispersing medium for the analyses. This phisical characterization was provided before and after light exposure in accelerated conditions. Scanning electron microscopy (SEM; Zeiss, Milan, Italy) was used to examine the morphology of the SLNs.

The thermal properties of pristine CB and DS, drug-free SLNs, and DS-loaded SLNs were analyzed using a Pyris Diamond Differential Scanning Calorimeter (PerkinElmer, Shelton, CT, USA) equipped with an intracooler refrigeration system. Samples (15–20 mg) were enclosed in small aluminum-foil disks and loaded at 50 °C. Then they were subjected to a cooling (20 °C)- heating (65 °C)- cooling (0 °C)- heating (65 °C)- cooling (−20 °C)- heating (65 °C) cycle at a scanning rate of 10 °C min^−1^. The sample temperature was held for 10 min at the start temperature of each cycle to allow slow relaxation/equilibrium of the sample, ensuring a stable and uniform starting state. An empty small aluminum-foil disk was used as a reference, and the baseline subtraction was applied to minimize the curvature of the measurement baseline.

Drug-lipid interactions and long-term stability of the formulation were tested by Fourier transform infrared spectroscopy (FTIR, Spectrum Two, Perkin Elmer, Milan, Italy). The instrument is equipped with a diamond crystal cell attenuated total reflection accessory (HATR top plate fitted with a 50 mm ZnSe crystal). The samples were placed on the ATR surface and infrared spectra were recorded between 4000 and 450 cm^−1^. All spectra were acquired at a resolution of 4 cm^−1^ and 32 scans.

### In Vitro* Drug Release Studies*

In vitro drug release studies of DS from CB-based SLNs were performed using a filtration cell equipped with a regenerated membrane (cut-off of 30 kDa) under constant stirring. The lipid formulation, with pH adjusted to 7.0, was dispersed in 50 mM phosphate buffer (pH 7.0). Drug release was evaluated at both 25 and 37 °C by collecting samples at predetermined time intervals and replacing the withdrawn volume with fresh buffer. Samples were analyzed spectrophotometrically (272 nm) to determine the amount of drug released. The experimental data were fitted to various kinetic models, including zero-order, first-order, Higuchi, and Korsmeyer–Peppas models, using linear regression analysis to determine the drug release mechanism.

### Photostability Test

Stability studies were performed on DS in aqueous solution prepared by using standard solutions of 20.0 mg mL^−1^. Since DS is poorly soluble, the drug solution was prepared in methanol. This sample was diluted in water until to obtain a concentration value of 20.0 μg mL^−1^ to perform photodegradation experiments. During the experiments, absorption spectra were registered in the range of 200–450 nm in a 10 mm quartz cell by means of a PerkinElmer Lambda 40P Spectrophotometer (PerkinElmer, Waltham, MA, USA) at the following conditions: scan rate 1 nm s^−1^; time response 1 s; spectral band 1 nm. The software UV Winlab® 2.79.01 (PerkinElmer, Waltham, MA, USA) was used for spectral acquisition and elaboration. According to the ICH rules, light exposure was simulated in the Suntest CPS + (Heraeus, Milan, Italy), equipped with a Xenon lamp. The irradiance power was 450 W m^−2^, corresponding to 27 kJ m^−2^ min, at a constant temperature of 25 °C. UV spectrum was measured just after preparation (t = 0 min) of the samples and at the following exposure times: 1, 3, 5, 10, 15, 20, 30, 40, 60, 90, 120, 150, 180, 210, 250 min. All chemometric procedures were made with the Matlab® computer environment software (Mathwork Inc., version 7, Natick, MA, USA). The MCR algorithm was applied to the spectral data, as average of six experiments, to calculate the drug concentration profile in the samples and to estimate the number of components, their spectra and the rate constants (k) of the kinetic processes [32, 34].

In a second step, photodegradation experiments were performed on DS entrapped in the SLNs formulations prepared as described above. In this case, during the light exposure, the spectrophotometric measurements were carried out at each time interval by diluting the sample with methanol. In the starting sample, the DS content was about 10.0 μg mL^−1^. As control, the solution containing DS and melted CB (macro mixture before MN), was exposed to light in the same accelerated conditions.

To minimize DS photodegradation, all laboratory experiments were carried out in a dark room under the illumination of a red lamp (60 Watt), kept at a distance of about 2 m.

## Results and Discussion

### Conventional Batch Nanoprecipitation

Conventional batch nanoprecipitation was used to identify the optimal conditions for the production of SLNs in the ouzo-stable region. This approach is commonly used to fine-tune key parameters, such as the solvent/non-solvent ratio and solute concentration, which directly influence particle size, polydispersity, and stability. As with polymeric materials, both the solute concentration and the solvent/non-solvent ratio play a crucial role in lipid nanoprecipitation, since these parameters govern the rate of supersaturation that drives the nucleation. In particular, the supersaturation rate affects particle size, as higher supersaturation rates lead to smaller particles, while lower rates result in larger ones [37].

**Fig. 2:**
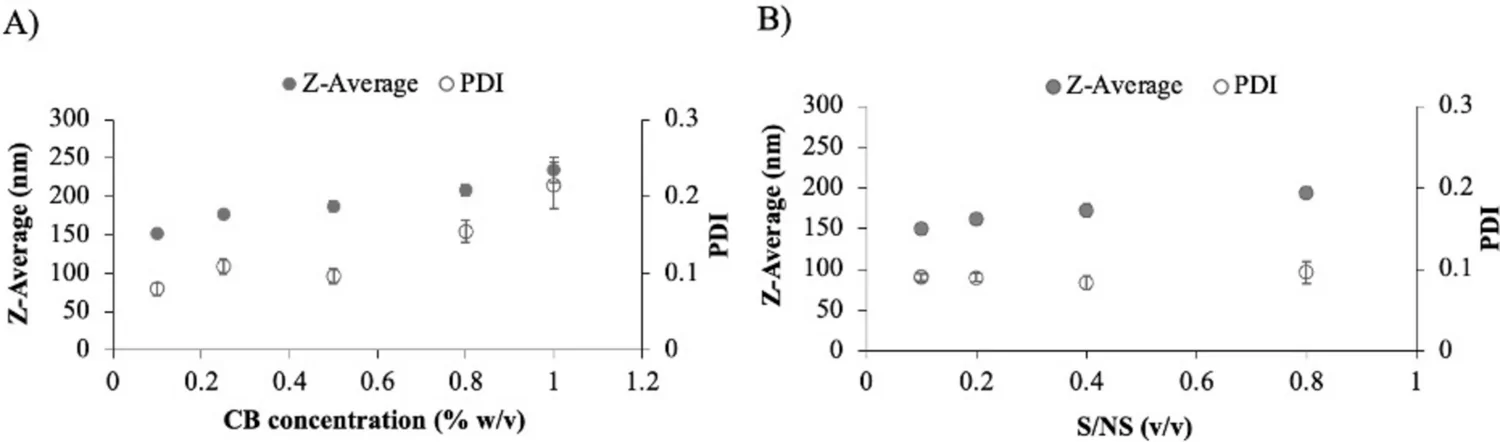
Effect of CB concentration at a constant solvent/non-solvent (S/NS) volumetric ratio of 0.2 **A** and of solvent/non-solvent volumetric ratio at a fixed CB concentration of 0.1% w/v **B** on the Z-Average and PDI of SLNs produced by conventional batch nanoprecipitation

The effect of CB concentration on the Z-Average and PDI (Fig. 2a) was evaluated by varying the lipid concentration from 0.1 to 1% w/v, while keeping the solvent/non-solvent volumetric ratio fixed at 0.2. The size of the SLNs increased from 150 to 235 nm with increasing CB concentration without significantly affecting the size uniformity, as evidenced by the low PDI value. The increase in size can be attributed to the higher viscosity of the lipid solution, as has also been observed in the nanoprecipitation of polymers [38]. The higher viscosity of the lipid solution slows down the diffusion rate of the ethanol into the non-solvent, resulting in slower supersaturation. This causes the growth rate to dominate over the nucleation rate. A PDI just above greater than 0.2 was achieved only with a CB concentration of 1% (w/v) where less control over nanoparticle formation resulted in particle aggregation. Similarly, particle size increased as the solvent/non-solvent volumetric ratio increased from 0.2 to 0.8, while the CB concentration remained constant at 0.1% w/v (Fig. 2b). As the concentration gradient between the two phases decreases, the exchange of solvent and non-solvent slows down, leading to a lower supersaturation rate and the formation of larger particles [39]. However, particle size uniformity with PDI less than 0.2 was always obtained by increasing the solvent/non-solvent volumetric ratio up to 0.8 (Fig. 2b).

### Membrane Nanoprecipitation

MN process has been widely studied for the production of polymeric NPs, but there is a lack of information regarding its use in the production of SLNs. The potential of MN for SLNs production was explored by evaluating the effect of the solvent/non-solvent volumetric ratio and fluid dynamic parameters on CB nanoprecipitation at a fixed CB concentration (0.1% w/v).

**Fig. 3:**
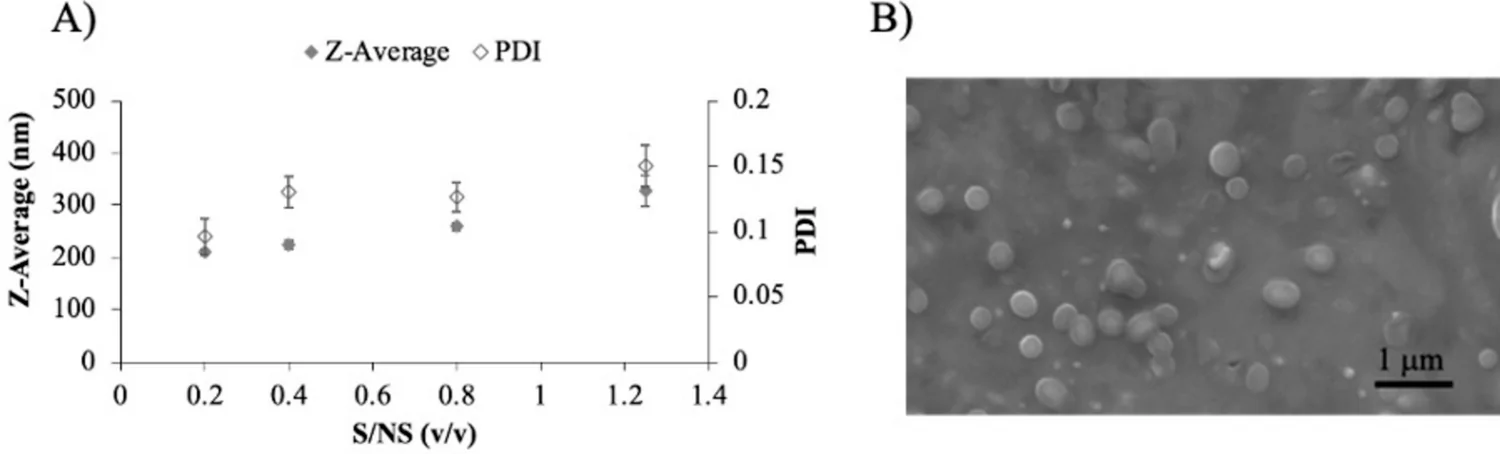
**A**) Effect of solvent/non-solvent (S/NS) volumetric ratio on Z-Average and PDI of SLNs produced by MN (CB = 0.1% w/v); **B**) SEM image of SLNs (MN, CB = 0.1% w/v, S/NS = 1.25)

Figure 3a shows the effect of the solvent/non-solvent volumetric ratio on particle size using 0.1% w/v CB. As with conventional batch nanoprecipitation, an increase in particle size was observed with increasing solvent/non-solvent volumetric ratio. In particular, particle size increased from 210 to 260 nm as the ratio of the two phases was varied from 0.2 to 0.8 (v/v).

Conversely, when the volume fraction of solvent in the mixture was significantly increased, the particle size increased up to 370 nm at a solvent/non-solvent volumetric ratio of 1.25.

It is notable that although the size increased, the uniformity of the formulation was not compromised and the PDI was always lower than 0.2 in all measurements. The SEM image of SLNs showed spherical particles in the nanometer range (Fig. 3b) with a quite uniform size distribution. The mean particle size obtained from the SEM measurements was around 350 nm, confirming the results obtained from the light scattering analysis (solvent/non-solvent = 1.25). Compared to the batch nanoprecipitation, the SLNs prepared by MN exhibited larger particle sizes (Fig. 2b), (i.e., at the solvent/non-solvent volumetric ratio of 0.8, the size of SLNs was 195 nm and 260 nm when batch nanoprecipitation and MN were used, respectively). This result has also been observed previously in MN for the production of polymeric and protein NPs [40, 41] and liposomes [19], as a result of the influence of the membrane on the mixing time between the organic solvent and the aqueous phase to achieve homogeneous supersaturation. This is a distinguishing aspect of MN that is confirmed to promote uniform nucleation and controlled particle growth, preventing aggregate formation or uncontrolled growth, also for the production of SLNs. Although the particle size obtained through the MN method is larger than that obtained with the conventional technique, MN results in continuous production of NPs. This is because the lipid solution is added to the non-solvent simultaneously through the membrane pores, thereby achieving a specific solvent/non-solvent ratio. In conventional batch nanoprecipitation, on the other hand, the lipid solution is added dropwise to the non-solvent over time to obtain a fixed solvent/non-solvent ratio.

**Fig. 4:**
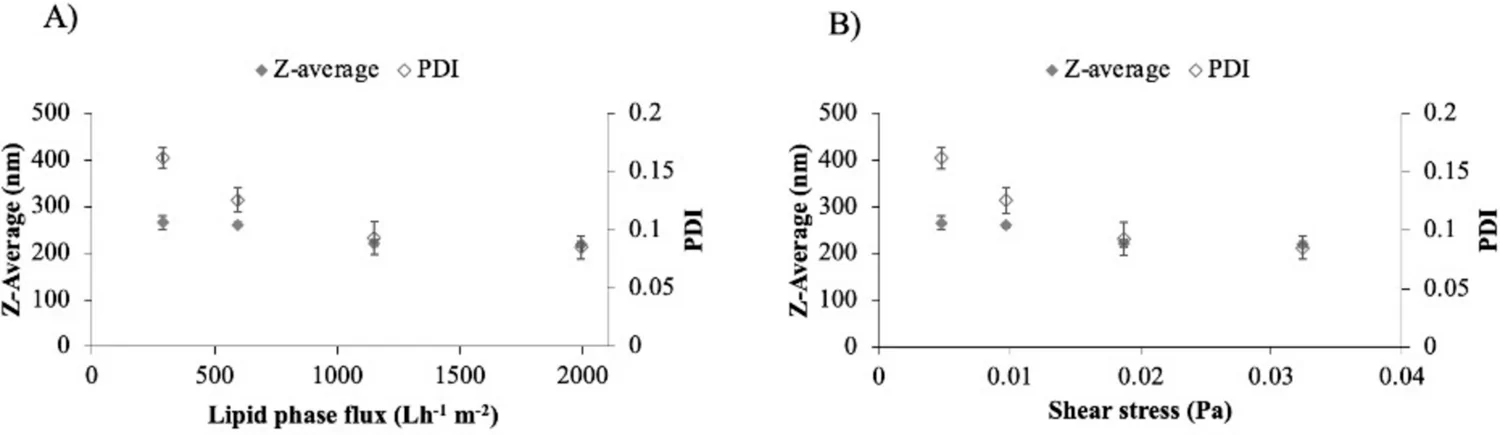
Effect of lipd phase flux **A** and shear stress **B** on the Z-Average and PDI of SLNs produced by MN (CB = 0.1% w/v, solvent/non-solvent volumetric ratio = 0.8)

To demonstrate the potential application of MN in the scalable production of the SLNs, the flux of the lipid phase was increased (Fig. 4a). Previous works on polymeric NPs and liposomes [40, 42] demonstrated that the particle size and particle size distribution obtained by MN were not significantly affected by the flux of the organic phase. Herein, a slight decrease in particle size (from 260 to 222 nm, with the PDI remaining below 0.2) was observed when the flux increased from 288 to 1994 Lh⁻^1^ m⁻^2^, suggesting an improved mixing of the two phases with increasing flux, as observed in static mixers [43]. In order to maintain a constant solvent/non-solvent volumetric ratio, the flow rates of both the lipid and the non-solvent phases were increased simultaneously. Specifically, increasing the non-solvent flow rate from 19 to 130 mL min⁻^1^ resulted in a corresponding increase in maximum shear stress from 0.005 Pa to 0.032 Pa.

These results indicate that MN enables increased mass productivity under low shear stress without negatively impacting particle size and particle size distribution, which is a significant advantage for the scale-up of the productive process (Fig. 4b). The MN productivity increased from 0.9 to 6.2 g h⁻^1^ as the organic phase flow rate increased from 15 to 104 mL min⁻^1^ (corresponding to a flux increase from 288 to 1994 L·h⁻^1^·m⁻^2^), using a membrane surface area of 31.3 cm^2^.

MN is a versatile method, as different configurations can be used to compartmentalize the two phases (solvent and non-solvent) within the membrane module (lumen and shell) (Fig. 1b).

Nanoprecipitation occurred either in the lumen or in the shell, depending on whether the CB-containing solvent phase permeated into the non-solvent phase or vice versa.

**Fig. 5:**
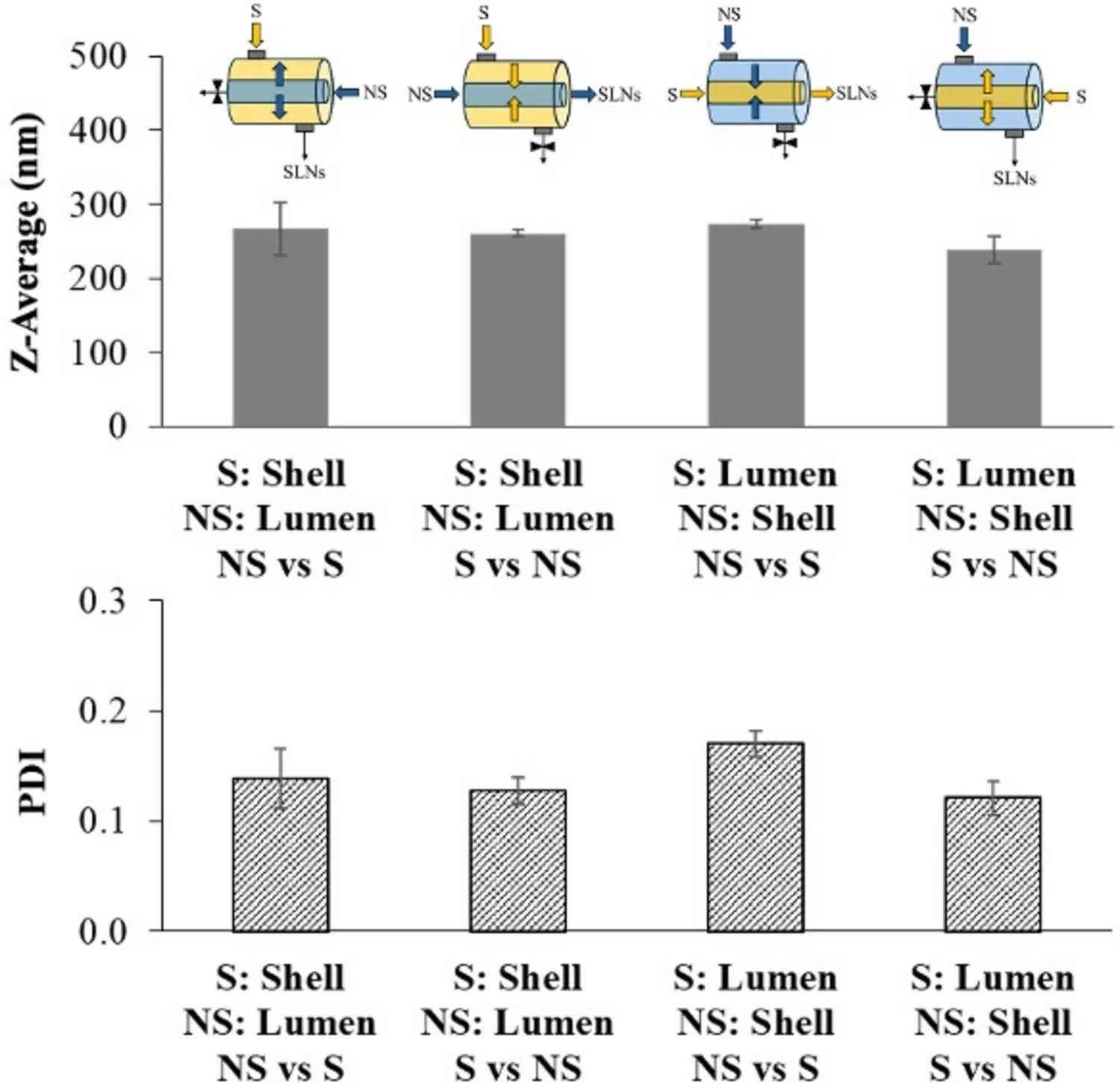
Effect of MN configurations on Z-Average and PDI of SLNs (CB = 0.1% w/v, solvent/non-solvent (S/NS) volumetric ratio = 0.8)

Once the organic phase and the aqueous solution met at the pore level, nanoprecipitation was promoted, regardless of the configuration used to compartmentalize the two phases. The configuration most commonly used for MN was one in which the solvent containing the solute permeated from the shell side to the lumen side [44, 45], while the permeation of the non-solvent into the solvent from the shell to the lumen has been reported for the production of protein-based particles [41, 46]. In all these cases, the nanoprecipitation occurred in the lumen of the membrane. The nanoprecipitation was only implemented in the shell for the production of drug nanocrystals in hollow fiber membranes [47–49] but has never been reported for tubular membranes. The influence of the different methods of adding one phase to another in tubular membrane was evaluated in this study (Fig. 5).

The data indicate that particles of comparable size can be obtained regardless of the configuration used. However, nanoprecipitation occurring in the lumen provides well-controlled particle size, as evidenced by the lower standard deviation observed. In particular, when the solvent or non-solvent permeated from the shell side to the lumen side, the size of the resulting SLNs exhibited a lower standard deviation (± 5 nm), compared to those obtained when CB nanoprecipitation occurred in the shell (standard deviation of ± 18 and ± 36).

This improved reproducibility of particle size can be attributed to the enhanced hydrodynamic control of the solvent and non-solvent mixing in the tubular lumen of the membrane considering that in the lumen a more defined flow is realized. Nevertheless, the PDI was less than 0.2 in all configurations, indicating good homogeneity of particle size.

### *Chemical-Physical Characterization and *In Vitro* Drug Release of the Proposed Formulation*

The DS-loaded SLNs were prepared by conventional batch nanoprecipitation and MN methods in order to evaluate the ability of the CB matrix to enhance DS photostability. A solution of DS (0.1% w/v) and CB (1% w/v) in ethanol was used as the solvent phase, while water or acetate buffer (pH 4.1) served as the non-solvent phase. The DS-loaded SLNs were formulated with a solvent/non-solvent volumetric ratio of 0.2.

**Fig. 6:**
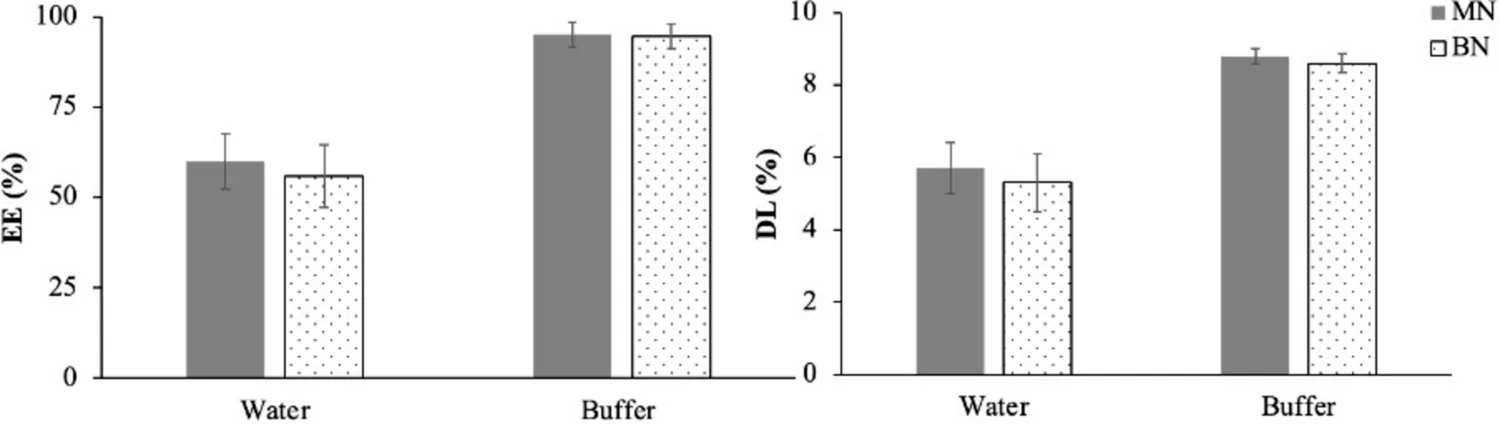
EE and DL in SLNs prepared by MN and conventional batch nanoprecipitation (BM) with DS = 0.1% w/v, CB = 1% w/v, and solvent/non-solvent volumetric ratio = 0.2

When pure water was used as the non-solvent phase, both methods produced an EE of 60 ± 8% and a DL of 5.7 ± 0.7% (Fig. 6). This EE value is higher than those reported in the literature (40.8–46.3%) for SLNs prepared with other types of lipids using the emulsion/solvent evaporation method [25]. The results showed that, although the logP value of DS (0.7) indicates a moderate affinity for the aqueous phase [50], a satisfactory EE was achieved. This could be due to the presence of unsaturated fatty acids in CB that can reduce the crystallinity of the lipid matrix [50], leading to structural disorder and enhancing the encapsulation capacity of DS. Furthermore, the lower crystallinity of CB compared to other lipids can be advantageous, as it minimizes the risk of drug expulsion during solidification, which is a common issue with highly crystalline lipid matrices [20].

A limitation of the nanoprecipitation method is the low EE of water-soluble molecules in NPs due to the rapid diffusion of molecules into the aqueous phase during the mixing of the two phases. Several strategies were employed to increase the EE of drug in NPs, such as incorporating of salts, pH adjustment, and solvent selection [51]. Among these strategies, considering the pH-dependent solubility of DS, the pH of the non-solvent phase was varied to increase the EE of DS within SLNs. Water was replaced with acetate buffer at pH of 4.1 as a non-solvent considering that the solubility of DS depends on the pH of the dissolution medium. In particular, the DS solubility is low in an acidic environment as the drug at a pH equal to its pKa (4.1) exists in equilibrium between ionized and unionized forms [52], resulting in a reduced solubility in aqueous solutions (DS solubility of 3.3 µg mL^−1^ at pH 4.1) [53]. The use of acetate buffer at pH 4.1 as the non-solvent phase led to enhanced DS entrapment, with an EE and DL of 95 ± 3.2% and 8.8 ± 0.2%, respectively.

Similar results were obtained by Liu et al. [26] with sodium DS-loaded SLNs prepared by emulsion/solvent evaporation method. In this study, a mixture of glycerol monostearate and phospholipid (Epikuron 200) served as the lipid matrix, while acidified water containing emulsifying agents was used as the dispersed phase. Moreover, the pH had no significant effect on the particle size of the DS-loaded SLNs. Comparable particle sizes of 416 ± 16 nm and 421 ± 14 nm were obtained by MN using water or acetate buffer as the non-solvent, respectively. The lipid formulation exhibited a zeta potential of −41.2 ± 2.3 mV, indicating good particle stability. Generally, zeta potential values greater than +30 mV or less than −30 mV suggest that particles carry sufficient surface charge to generate strong electrostatic repulsion, thereby preventing aggregation and promoting dispersion stability. In view of these results, optimizing the pH conditions during nanoparticle preparation is crucial for maximizing DS encapsulation. The SLNs produced with the highest EE were then used to study the photostability of the DS.

The thermal behavior of pristine CB and DS, as well as drug-free SLNs and DS-loaded SLNs was analyzed using differential scanning calorimetry (DSC).

**Fig. 7:**
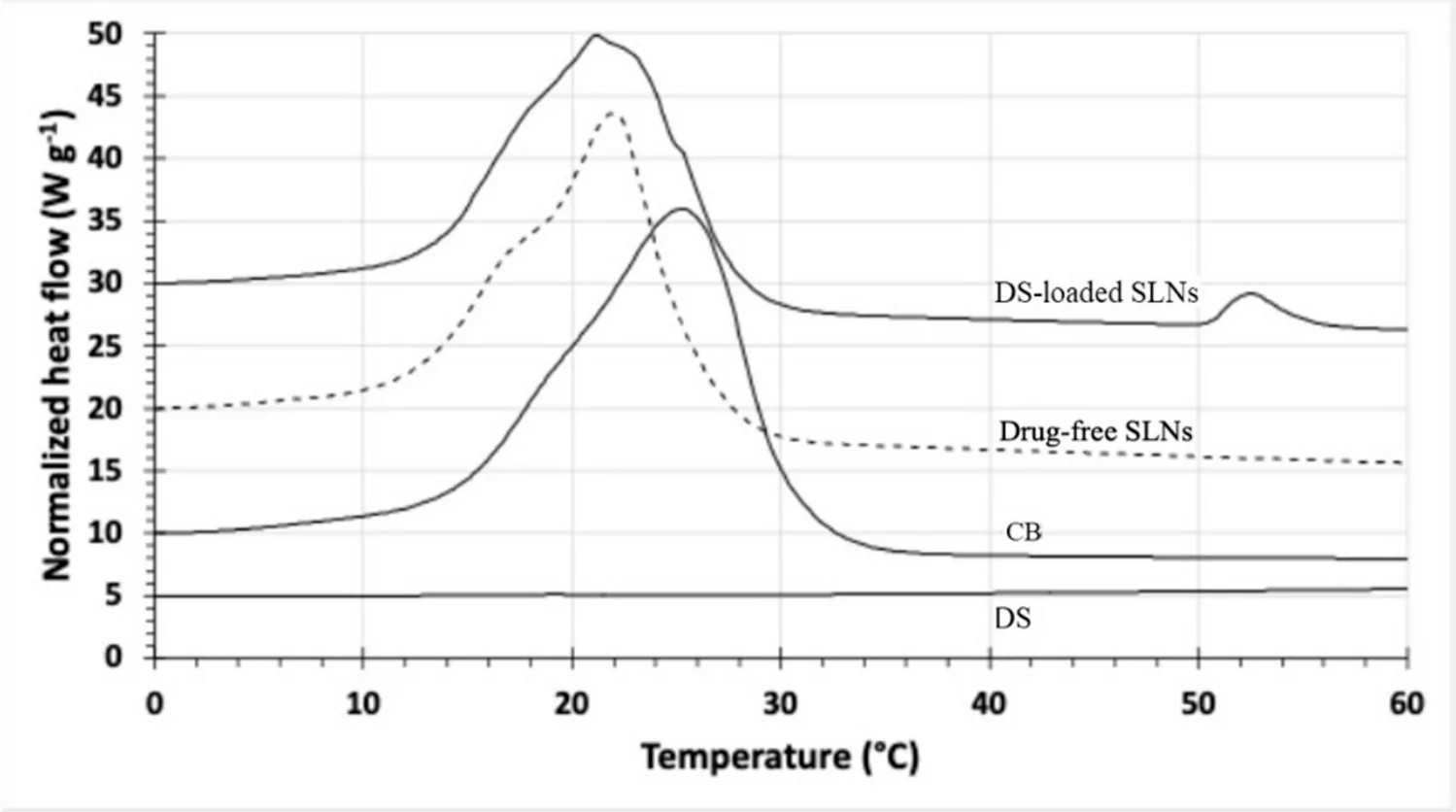
DSC thermograms during the third heating cycle for DS, CB, CB SLNs (drug-free) and DS-loaded SLNs. Curves are shifted vertically to improve readability

The results of the second and third heating runs of the DSC analysis and in overlaps, showed that the conditioning at 65 °C after the first heating run was necessary to delete any previous thermal history [54], together with the long conditioning time. The third heat runs of all the samples are reported in Fig. 7, which for the pristine CB shows a wide endothermic peak with a maximum at about 26 °C due to the fusion of the various crystal types present in pristine CB. The presence of different crystal types is supported by the shoulder present at about 19 °C. A single endothermic peak appears also for the CB-based SLNs after nanoprecipitation. However, this peak shape is sharper with respect to the pristine CB and the maximum is reached at lower temperature (approximately 22 °C), showing a change in the polymorphs type in the CB when it is in NPs form. This melting behavior is consistent with that observed in previous studies of CB-based SLNs prepared through emulsification method employing hot high-pressure homogenization [10].

A quite different DSC-thermogram appears when DS is embedded in the SLNs, which reports two endothermic peaks. One peak can be ascribed to the fusion of the polymorphs of CB, and the maximum at approximately 21 °C shows that there is a prevalence of the same main polymorphs present in the CB-based SLNs after nanoprecipitation. However, in this case the peak is much wider at higher temperatures, showing that the presence of DS influences the crystals formation. Even more interesting is the small endothermic peak that appears at 52–53 °C and that is not present neither in the CB, nor in the DS thermogram (that is totally flat in the investigated temperature range, in agreement with literature [55]. This peak can be ascribed to the formation of an inclusion complex between the CB as host and the DS as guest, mainly driven by non-covalent interactions. A full characterization of the formation of the inclusion complex would require also the analysis of the fusion peak of the DS. Unfortunately, this analysis cannot be performed on this system since the melting point of the DS is at about 280 °C [55] and the CB is not stable in that temperature range.

**Fig. 8:**
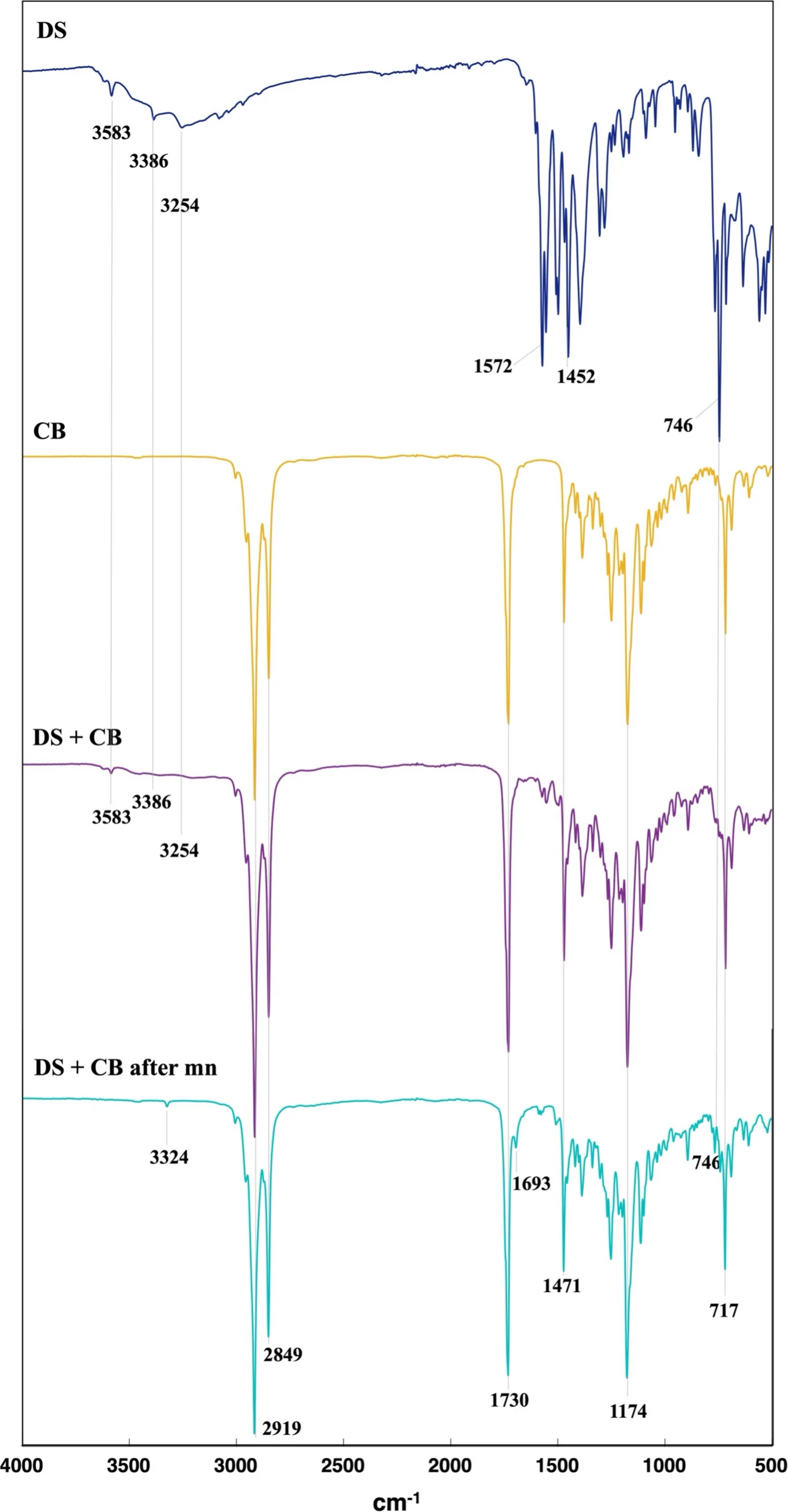
Overlapping of FTIR spectra of DS, CB and their mixture before and after MN. Major peaks typical for each compounds and present in the mixture are marked

The entrapment of the drug in the SLNs was demonstrated by FTIR. The typical peaks for each compound were detected in their mixture spectrum measured before and after the nanoprecipitation procedure, confirming the absence of chemical interactions between DS and CB. The overlapping of the FTIR spectra of DS, CB and their mixture before and after MN is shown in Fig. 8. The spectrum of DS showed the intense, well defined infrared band at around 1572 cm^−1^ (C = O stretching of the carboxyl ion), 1452 and 746 cm^−1^ (C-H bend and C–Cl stretching, respectively) [56]. The peaks at 3254 and 3583 cm^−1^ (O–H stretching vibrations) and 3386 cm^−1^ (NH stretching of the secondary amine) were also observed.

According to the literature [57], the FTIR spectrum of CB shows peaks including 2914 cm^−1^ and 2849 cm^−1^ for CH₂ stretching, a strong band for the ester carbonyl (C = O) at 1730 cm^−1^, and other peaks related to C-H bending and the glycerol backbone. There is no significant change in the absorption spectra for the drug when prepared in mixture. Due to the use of acetate buffer at pH 4.1 as the non-solvent phase during MN procedure, the drug is no longer present in salt form but in free acid form. Therefore, the spectrum of the mixture after MN (which resulted in the formation of the DS-loaded SLNs) shows a specific absorption peak at 3324 cm^−1^, which corresponds to free OH stretching of a carboxylic group and a peak at 1693 cm^−1^ associated with C=O stretch [58].

A long-term stability test was assayed by FTIR four months from preparation confirming the spectrum of the prepared formulation and thus the stability of the SLNs over time. Sample formulation was maintained at room temperature in a dark room.

**Fig. 9:**
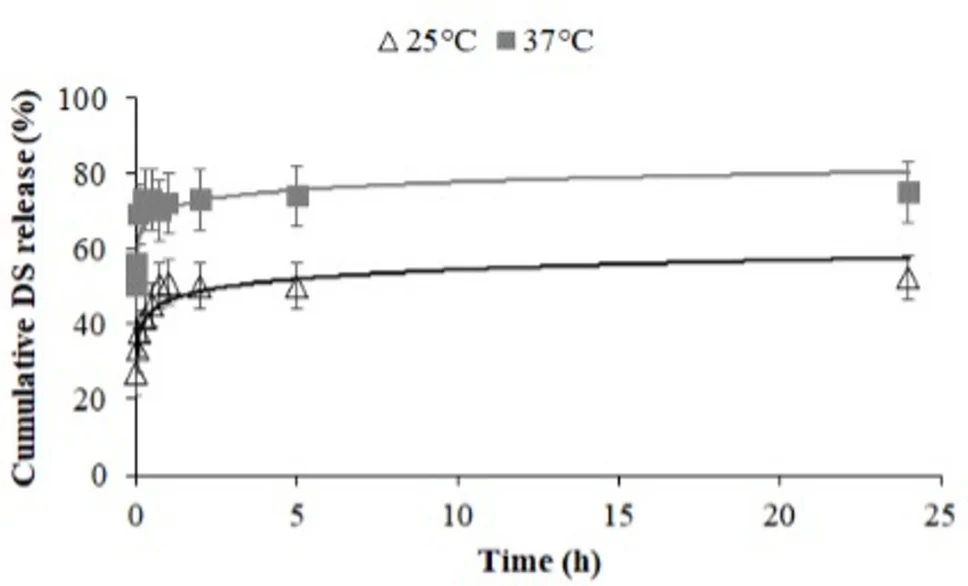
Cumulative in vitro release of DS from CB-based SLNs at 25 °C and 37 °C as a function of time

Figure 9 shows the cumulative release of DS from CB-based SLNs over time at 25 °C and 37 °C. The drug release is clearly temperature-dependent, with a more pronounced release is observed at 37 °C, where over 50% of DS is released within the first minutes, followed by a sustained release reaching about 75%. This behaviour can be attributed to the melting of the CB matrix at 37 °C, which reduces its structural integrity and enhances drug diffusion into the surrounding medium. Therefore, the release process is likely governed not only by diffusion but also by matrix phase transition. In contrast, at 25 °C, the release is slower and reaches a plateau at approximately 50%. The melting point of CB close to that of human body results to be particularly useful for topical applications as the contact with the skin (37 °C) can promote a significant release rate increase, ensuring that the active ingredient becomes available only when needed.

Among the evaluated kinetic models (Table 1), the Korsmeyer–Peppas model provided the best fit to the experimental data, exhibiting the highest regression coefficients at both temperatures (R^2^ = 0.9336 at 25 °C and 0.9411 at 37 °C). This indicates that it is the most appropriate model to describe the drug release behaviour from the SLNs. The release exponent (n) was < 0.5 at both temperatures, suggesting that drug transport is predominantly governed by Fickian diffusion through the lipid matrix.

**Table 1:** Kinetic parameters of in vitro DS release from CB-based SLNs

Model	Temperature (°C)	Release constant (K)	Release exponent (n)	Regression coefficient (R^2^)
Zero order	25	0.98	–	0.7375
	37	1.62	–	0.7327
First order	25	0.34	–	0.7647
	37	1.00	–	0.7744
Korsmeyer-Peppas	25	0.57	0.169	0.9336
	37	0.94	0.146	0.9411
Higuchi	25	0.40	–	0.8663
	37	0.62	–	0.8601

### *The Greenness of the MN* Process

The sustainability of the developed MN process was evaluated by performing a quantitative assessment of energy consumption and green metrics (sEF and cEF) (Tables 2 and 3).

**Table 2:** Productivity and energy consumption in the MN process

CB concentration (% w/v)	Solvent (S) flow rate (mL min^−1^)	Non-Solvent (NS) flow rate (mL min^−1^)	S/NS ratio	Productivity (g h⁻^1^)	Energy consumption	
					J m^−3^	W m^−3^
0.1	15.2	19.0	0.8	0.9	2.8 × 10^3^	46.84
	31.2	39.0		1.9	5.8 × 10^3^	95.80
	60.0	75.0		3.6	1.1 × 10^4^	189.25
	104.0	130.0		6.2	1.9 × 10^4^	312.50
1	31.0	155.0	0.2	18.6	3.8 × 10^3^	62.50

**Table 3:** Green metrics assessment of the production of DS-loaded SLNs by the MN process

Green metrics	Type of non-solvent for MN process	
	*Pure water*	*Acetate buffer (pH 4.1)*
sEF	0.038	1.52
cEF	546.17	520.20
% cEF	6.9 × 10^–5^	2.9 × 10^–3^
% solvent + water	99.81	99.53

The low shear stress used during the nanoprecipitation process (0.032 Pa) offers the advantage of lower energy consumption during nanoparticle production without significantly modifying the particle size distribution of the lipid formulation. This result is in agreement with previous data reported for the production of polymeric particles. It is notable that the MN method permits the production of lipid NPs while consuming less energy compared to conventional methods [36].

In particular, the energy consumption associated with the production of lipid NPs by high-pressure homogenizers is reported to reach a maximum value of 10^12^–10^13^ W m^−3^ [59], which is considerably higher than the maximum value used in the present work (312.50 W m^−3^). If we consider that the productivity of the process can be increased up to 18.6 g h⁻^1^ (by using 1% w/v CB) while maintaining a low energy consumption (3.8 × 10^3^ J m⁻^3^ or 62.50 W m⁻^3^), it is possible to underline that the use of the MN process permits an increase in productivity with minimal energy input.

Green metrics, such as sEF and cEF, are widely used to assess the environmental impact of chemical syntheses and have more recently been applied to evaluate the sustainability of membrane technology for the production of polymeric particles [60].

This analysis was extended to the production of SLNs by MN (Table 3). According to the sEF, which quantifies the materials consumed (excluding water and solvent), the use of acetate buffer instead of water increased the sEF from 0.038 to 1.522. However, a more comprehensive analysis is required to evaluate the relevance of this value. The improved EE itself contributed to the sustainability of the process by reducing the amount of non-encapsulated DS, which would otherwise require additional treatments to mitigate its environmental impact [61]. Moreover, it reduces production costs, as the drug is one of the most expensive materials used in particle manufacturing [60]. Conversely, the buffer components are inexpensive and non-toxic. It is notable that the production of free-emulsifying SLNs by MN further avoids the generation of additional waste.

Considering the contribution of the solvent and the water, the use of equal volumes of ethanol and either pure water or acetate buffer in both strategies resulted in comparable cEF values as for % solvent + water.

A further contribution to the sustainability of the SLNs production process can be achieved by integrating the MN process with a UF unit to enable complete recovery of the SLNs and their purification from the solvent mixture. A proof-of-concept of this strategy was provided at small-scale production using a centrifugal filtration device. The test demonstrated that the UF membrane can effectively recover the SLNs. After the UF step, particle size, zeta potential and encapsulation efficiency remained almost unchanged (Table 4).

**Table 4:** Z-average, zeta potential, and encapsulation efficiency of DS-loaded SLNs before and after the UF operation

DS-loaded SLNs	Z-Average (nm)	Zeta potential (mV)	Encapsulation efficiency (%)
Before UF	421 ± 14	−41.2 ± 2.3	95.0 ± 3.2
After UF	442 ± 24	−40.4 ± 2.2	93.4 ± 4.1

A clear permeate solution was obtained after the UF operation, indicating that all SLNs were efficiently retained in the retentate. This result was further confirmed by light scattering analysis of the permeate, which did not reveal any distinct particle populations.

Overall, the process does not generate waste requiring disposal, as the ethanol can be recovered by the UF process (which can be subsequently purified by using membrane distillation to be reused in subsequent production cycles). This integration could make the overall process energy-efficient and environmentally sustainable. Due to the scalability of membrane processes, this approach can be easily implemented for treating larger volumes.

### Photostability Studies

Lipid-based solid carriers present promising advances in drug delivery systems, which favor their clinical application. In addition to influencing their therapeutic efficacy, the developed formulations could promote the physical stability of drugs by increasing their shelf-life. Thus, photodegradation experiments were planned according to the ICH rules. In our previous studies, the high sensitivity to light of the selected drug has been verified in methanol solution, standard gel and commercial gel specialty. The use of antioxidant molecules, UV-absorber agents and the incorporation of DS in cyclodextrins or niosomes have been shown to improve photostability [23, 24].

**Fig. 10:**
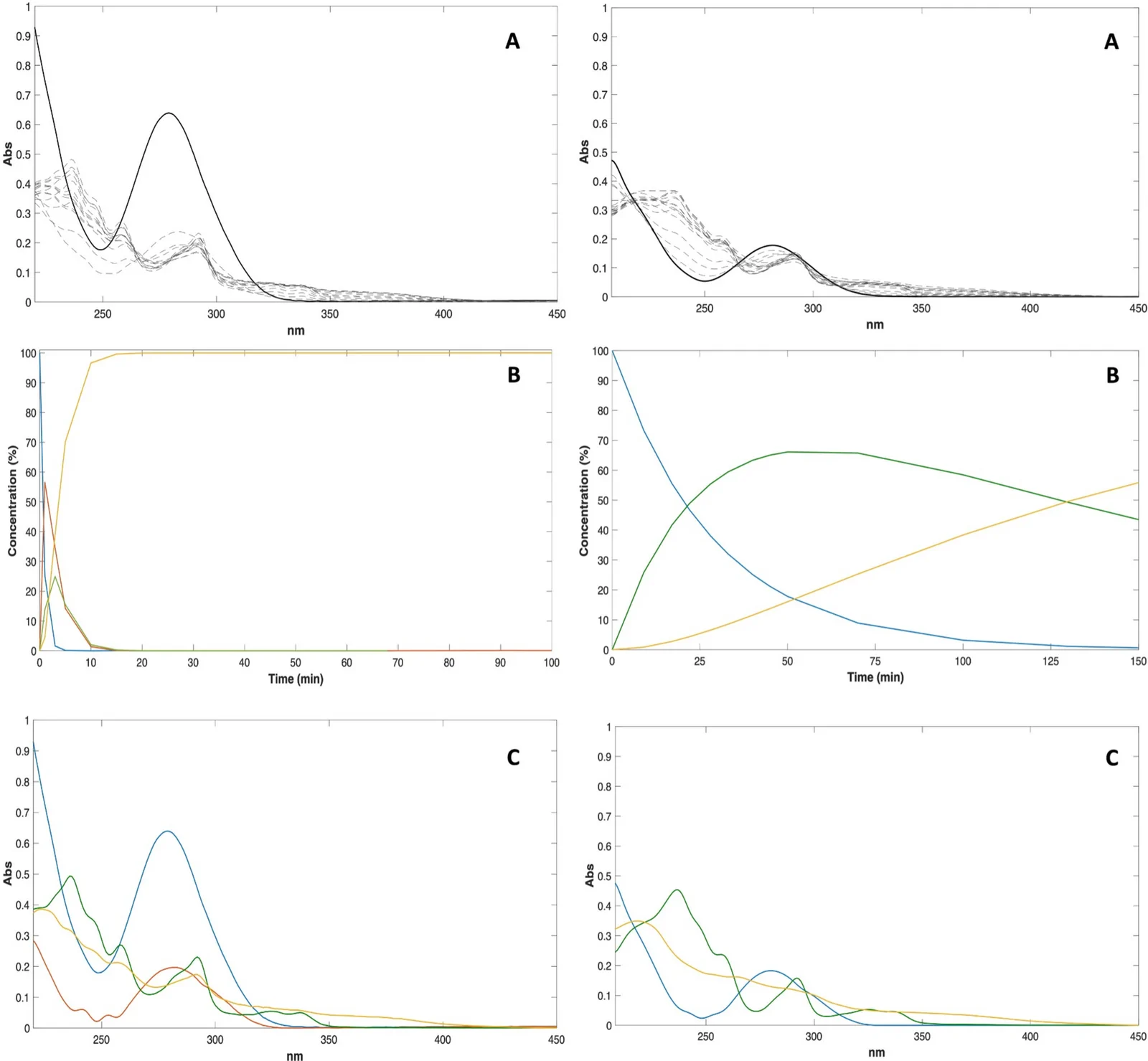
Absorbance spectra (Part A) of DS aqueous solution and DS-loaded SLNs, just after preparation (—) and after irradiation (••••); concentration profiles of DS and photoproducts (part B) and relative absorbance spectra from MCR elaboration when prepared in solution and in SLNs (part C). Blue line: DS pure compound; green line: ph1; yellow line ph2; red line: ph3

Herein, the light sensitivity of DS was studied in aqueous solutions and in the proposed SLN systems. As described in paragraph “Photostability test”, the experiments were performed by exposing the samples to light at different time intervals in the Suntest CPS+ cabinet. Photodegradation profiles were monitored by spectrophotometry and the obtained data were matched by chemometric approaches using MCR-ALS method. The multivariate resolution methods provide to decompose the data matrix from the photodegradation experiment into the pure contributions of the single components. In this way, the estimation of the correct number of species involved and their concentration profile was achieved. Figure 10a shows the sequence of the spectra recorded just after the preparation and at several exposure times for both the tested samples.

In all experiments, the degradation process followed first-order kinetics. The residual drug percentage (DS%) is correlated to k (rate constant) and t (time expressed in sec) according to the below reported equation where 4.61 is the logarithm (ln) of the starting concentration (100%):

$$ ln\left[DS\%\right]=-kt+4.61$$

The quality of the MCR results was evaluated by calculating the parameter lack of fit (%lof), which indicates that the model fits all the design points well and the R-Squared (R^2^ or coefficient of determination). In all experiments, %lof was less than 8% and R^2^ was higher than 99.4%. Figure 10 shows the concentration profiles of DS and photoproducts (part B) and their absorbance spectra (part C) when prepared in solution and in SLNs. The parameters t_0.1_ and t_0.5_ (time to cause 10% and 50% degradation, respectively) were selected to compare the degradation behavior of the tested samples. Table 5 lists kinetics parameters and t_0.1_ and t_0.5_ values calculated for the samples.

**Table 5:** Kinetic parameters calculated for DS samples

Sample	k	SD ± k	R^2^	t_0.1_ (min)	t_0.5_ (min)	%lof
DS aqueous solution	0.0228	0.0035	99.48	0.072	0.51	7.18
DS-melted CB	0.0195	0.0025	99.77	0.094	0.60	4.81
DS-loaded SLNs	0.0006	0.00005	99.99	3.34	19.45	6.45

In agreements with the results obtained in previous works for ethanolic solutions, when DS aqueous solution was exposed to light, the formation of one major photoproduct (ph1) and traces of the other two by-products (ph2 and ph3) was verified. DS residual concentration resulted to be 90% after a few second of exposure with a t_0.1_ value of 0.072 min and 50% after almost a minute of irradiance (t_0.5_ value of 0.51 min), whereas a full degradation was observed after about 2 min.

In the next step, photodegradation experiments were performed on DS in the proposed SLN formulations, prepared as described above. In this case, the photoprotection of the drug was evident. DS residual concentrations of 90% and 50% were calculated after 3.34 and 19.45 min of light exposure, respectively. The presence of the pure drug was still measured until to 110 min. Particle size measurements were also performed after stressing irradiation confirming physical stability of the nanoparticles during light exposure. Particle size analysis showed that the lipid formulation remained substantially stable over the investigated time range (up to 250 min), with a slight increase in mean particle size from 421 ± 14 (at the time of preparation) to 472 ± 30 nm (after 250 min of irradiation). Similar results in terms of particle size were obtained by analysing the formulation without any stressing irradiation at the same time (440 ± 25 nm) and after 10 days of storage (460 ± 27 nm).

MCR analysis (Fig. 10 part C) confirmed the formation of a main photoproduct and another product that converts into the first. Salgado et al. [62] proposed the formation of the decarboxylated product (E)−6-[2,6dichlorophenyl)-imino]−3-oxocyclohexa-1,4-dienecarbaldehyde when DS solution is exposed to light. UV spectrum of this compound correspond to the spectrum proposed in Fig. 10 (part B and C) as major photoproduct (yellow line). A few years later, Iovino et al. [10, 63] hypothesized a possible dimer formation pathway from the DS degradation by photolysis with the formation of a carbazole-1-acetic acid starting from an epoxide production, followed by the loss of both chlorine atoms. UV spectrum of the epoxide compound corresponds to the second by-product reported in Fig. 10 (part C) as secondary photoproduct (green line). Thus, we can postulate a photodegradation mechanism according to two consecutive reactions in which, starting from the pure compound DS, an epoxide intermediate is formed, which in turn is converted into the carbazole derivative.

DSC analysis revealed a distinct, small endothermic peak at 52–53 °C, which is absent in the individual components; this suggests the formation of an inclusion complex between the CB host and the DS guest, primarily driven by non-covalent interactions. The formulation maintains its chemical integrity, as confirmed by FTIR analysis which showed no evidence of covalent chemical interactions between DS and CB; the characteristic peaks of both compounds remain detectable, indicating that the drug is physically encapsulated without undergoing permanent structural alterations during the manufacturing process.

Consequently, the solid lipid matrix acts as a robust physical barrier that isolates the drug molecules from the external environment, effectively limiting both direct exposure to photons and contact with oxygen, thereby minimizing oxidative photodegradation. CB inherently possesses natural properties that allow it to absorb or reflect UV radiation, and when structured in the form of SLNs, it functions as an integrated protective layer. This photoprotective mechanism is further validated by the stress-testing of a mixture of DS and melted CB (prepared prior to membrane nanoprecipitation); while this mixture was more stable than a simple aqueous solution, it still degraded with a k and t_0.1_ value of 0.0195 and 0.094 min, respectively, as reported in Table 5. This confirms that while the presence of tocopherol in CB helps mitigate UV-induced degradation by acting as a free radical scavenger, the specific drug-lipid complexation and SLN architecture are far more effective strategies for maximizing photoprotection.

Finally, comparing the degradation profile of this SLN formulation with traditional methods involving antioxidant molecules highlights a significant advantage: while adding antioxidants is a straightforward approach to improve photostability, it does not enhance the drug's ability to penetrate biological barriers. In contrast, these SLNs are specifically designed to potentiate the transdermal and corneal permeation of DS. Although antioxidants act purely through chemical neutralization of free radicals, they lack the structural protection of a lipid matrix that physically shields the drug. Furthermore, CB-based SLNs provide additional moisturizing and emollient benefits that facilitate drug absorption and improve skin conditioning, serving as an advanced delivery platform that simultaneously enhances the bioavailability and therapeutic efficacy of DS benefits that antioxidants alone cannot provide.

## Conclusions

This study explored the use of MN for preparing SLNs with CB as the lipid matrix to encapsulate DS. The results showed that MN is an effective and sustainable method for continuous production of DS-loaded SLNs. Lipid anoprecipitation is primarily influenced by lipid concentration and phase ratio, rather than by organic flux and MN configurations. The formation of SLNs of similar size occurred in the lumen or shell following permeation of the non-solvent into the solvent (and vice versa) through the membrane pores.

The MN process permitted to achieve a productivity up to 18.6 g h^−1^ operating under mild shear stress conditions (0.040 Pa) and with low energy consumption (3.8 × 10^3^ J m^−3^). Moreover, the process can be optimized to improve the EE and DL of DS by adjusting the pH of the non-solvent phase. DS-loaded SLNs with a size of 421 ± 14 nm were produced with an EE of 95 ± 3.2% and a DL of 8.8 ± 0.2%, using acetate buffer at pH 4.1 as the non-solvent phase. In contrast, the use of pure water resulted in an EE and a DL of 60 ± 8% and 5.7 ± 0.7%, respectively. The MN proved to be a promising strategy for greening the scalable production of drug-loaded SLNs compared to conventional techniques. Satisfactorily, the efficacy of the SLNs in protecting the drug from photodegradation was also demonstrated with a stability increase of almost five times. The system should provide a valuable tool for the development of new pharmaceutical formulations able to improve drug viability and photostability.

## Data Availability

The datasets generated during and/or analysed during the current study are available from the corresponding author upon reasonable request.
